# PERCEPT: Replacing binary *p*-value thresholding with scaling for more nuanced identification of sample differences

**DOI:** 10.1016/j.isci.2024.109891

**Published:** 2024-05-03

**Authors:** Dezerae Cox, Danny M. Hatters

**Affiliations:** 1 Department of Biochemistry and Pharmacology, Bio21 Molecular Science and Biotechnology Institute, The University of Melbourne, Parkville, VIC 3010, Australia; 2 Yusuf Hamied Department of Chemistry, University of Cambridge, Cambridge CB2 1EW, UK; 3 UK Dementia Research Institute, University of Cambridge, Cambridge CB2 0AH, UK; 4 Molecular Horizons, School of Chemistry and Molecular Bioscience, University of Wollongong, Wollongong, NSW 2500 Australia

**Keywords:** Natural sciences, Biological sciences, Systems biology

## Abstract

Key to a biologists’ capacity to understand data is the ability to make meaningful conclusions about differences in experimental observations. Typically, data are noisy, and conventional methods rely on replicates to average out noise and enable univariate statistical tests to assign *p*-values. Yet thresholding *p-*values to determine significance is controversial and often misleading, especially for omics datasets with few replicates. This study introduces PERCEPT, an alternative that transforms data using an ad-hoc scaling factor derived from *p-*values. By applying this method, low confidence effects are suppressed compared to high confidence ones, enabling clearer patterns to emerge from noisy datasets. The effectiveness of PERCEPT scaling is demonstrated using simulated datasets and published omics studies. The approach reduces the exclusion of datapoints, enhances accuracy, and enables nuanced interpretation of data. PERCEPT is easy to apply for the non-expert in statistics and provides researchers a straightforward way to improve data-driven analyses.

## Highlights

• We introduce PERCEPT, a scaling method for noisy biological datasets
• PERCEPT reduces data loss, enhances accuracy, and enables nuanced interpretation
• PERCEPT is easy to use for biologists and especially well-suited to omics data

## Graphical abstract

**Figure:**
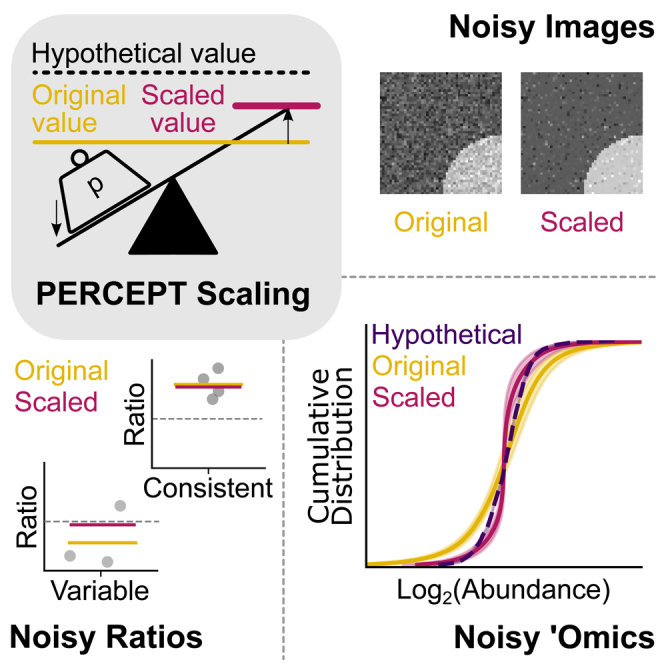

## Introduction

Variability in sample measurements is an unavoidable part of biological research.^1^^,^^2^ Variability can be measured using biological replicates, which in turn allows differences to be determined with univariate statistical tests that provide *p-*values. It has become the gold-standard to apply binary thresholding for assessing *p-*values, such as setting *p* < 0.05.

However, the use of statistical significance as a decision boundary is imperfect, and indeed controversial.^3^^,^^4^^,^^5^ Statistical inference is not equivalent to scientific inference, with scientific inference requiring further investigation.^6^^,^^7^ Yet, too often in the literature scientific conclusions are drawn just from binary thresholding of *p*-values.^5^ Furthermore, binary thresholding encourages poor practices, such as selective reporting of data that are “significant”, and “p-hacking” which describes scanning a range of statistical tests and/or data combinations to find a desired level of significance.^8^ Collectively these outcomes have reduced the reliability of research findings and have led statisticians to suggest the term “statistically significant” be replaced with nuanced, customized statistical strategies that treat *p-*values as a spectrum.^4^^,^^5^^,^^9^

To this goal, we present an approach to detect patterns in datasets that does away with binary thresholding of *p*-values and may be especially useful under conditions where binary thresholds perform poorly (i.e., datasets with low replicates and high variation). The method, P-value Enhanced Rescaling for Comprehensive Exploration and Preservation of Trends (PERCEPT), is designed to be easy to use for biologists and is well suited to omics datasets where it is important to identify patterns of associations in the genes/proteins that differ between samples. We report the application of PERCEPT to simulated and real data, and demonstrate its ability to closely align scaled data with the ground truth. This method is amenable to either simple spreadsheet- or code-based implementations, enabling easy integration of PERCEPT into current analysis workflows alongside traditional statistical testing.

## Results

**Figure 1:**
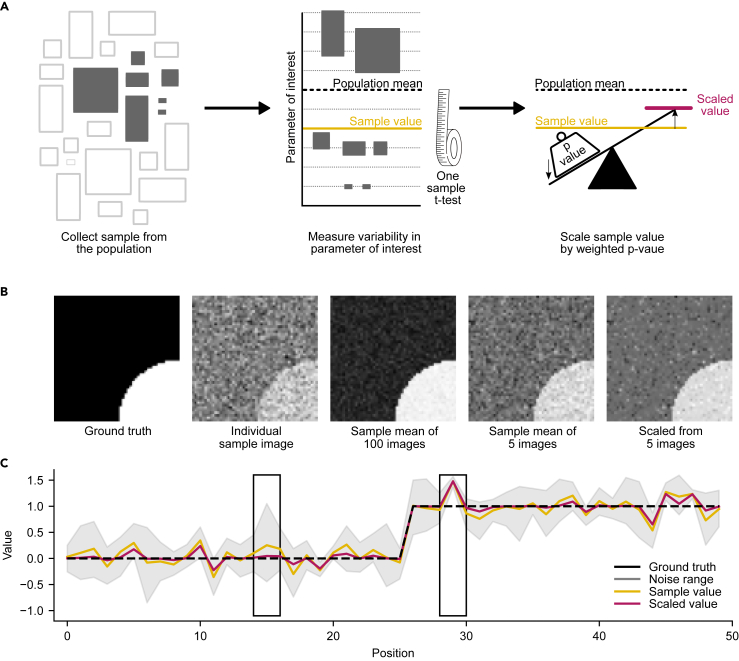
Implementation of PERCEPT scaling (A) Schematic of PERCEPT. In most biomedical studies, a sample of the population is measured for a parameter of interest. The statistical difference between the sample mean and hypothetical population mean can then be measured using a one-sample t-test. The derived *p-*value is then converted to an inverse scaling factor, effectively moving low-confidence measurements toward the hypothetical mean. (B) Comparison of large*-n* mean smoothing with small*-n* scaling on simulated image dataset. From a ground truth image consisting of zeros and ones, random noise was introduced to individual sample images. Taking the mean of large*-n* subpopulations (e.g., 100) largely recovers the true result. However, mean smoothing of small*-n* subsamples (e.g., 5 images) is outperformed by scaling with PERCEPT. The *p*-value was derived from a one-sample t-test against the ground truth value at each pixel coordinate, and the penalty factor used was 20. (C) Simulated line scan from B. The range of values for each individual pixel location across 5 sample images (noise range 95% confidence interval; gray bar) is shown. The ground truth value (black dotted) is compared to the mean (yellow) and scaled (magenta) values. Regions of high variability in the noised image (left box) are impacted by scaling while those with low variability in the noised image (right box) are not. See also Figure S1.

The gist of PERCEPT is that it uses *p-*values as a measure of confidence in the effect size (Figure 1A). To demonstrate how it works, we first consider a scenario where a *p-*value is obtained through a one-sample t-test which compares a mean sample parameter against a hypothetical value. This hypothetical value can be based on experimental or assumed knowledge of the true value, i.e., the ground truth. PERCEPT transforms the *p-*value into an ad-hoc inverse scaling factor which is applied to the sample parameter. As the *p-*value increases in value, the scaling factor brings that value closer to the ground truth and hence is useful for suppressing values of noisy low confidence data whilst preserving high confidence data. The intensity of this scaling can be adapted using a tunable penalty (F; Figure S1), which we define, arbitrarily, as 10×n. For example, consider a situation where the ground truth value was 600, and we measured a subset of the population that had a mean of 400. If the variation between subset data was high and of low confidence (e.g., a *p*-value of 0.5), then PERCEPT would bring that value to close to the ground truth (563 assuming n = 3, and thus *F* = 30, using Equation 1). If, on the other hand, the mean of 400 had a high confidence (e.g., a *p*-value of 0.02) then PERCEPT would preserve most of that measured value (413 assuming n = 3 using Equation 1). While not itself derived from frequentist statistics, PERCEPT provides a nuanced interpretation of *p-*values to deliver comprehensive understanding of the underlying effect sizes considering inter-replicate variability.

The impact of PERCEPT can be visualized using a simulated dataset (Figure 1B). Here, the ground truth data consists of a two-dimensional array, represented as an image, with zeroes and ones. When random noise, akin to biological variability, is added to each pixel position, the resulting images appear speckled (Figure 1B, individual sample image). Averaging many replicates (e.g., 100) minimizes the effect of this noise, resulting in images with reduced speckling (Figure 1B). In contrast, small sample sizes (e.g., n = 5) are less smoothed than n = 100 when averaged, as anticipated for noisy data (Figure 1B). PERCEPT, when applied to n = 5, reproduces an image closer to the ground truth than averaging (Figure 1B). The effect of PERCEPT compared to averaging is particularly pronounced when the underlying sample data show high variability (Figure 1C). For instance, in Figure 1C, line scans at a single y-coordinate in the simulated image illustrate the ground truth value at each x-coordinate overlaid with the range of noise in the small*-n* sample. In cases where the replicates exhibit high variability (Figure 1C, left box), scaling effectively smooths the value toward the ground truth. However, when the sample value consistently deviates from the ground truth, both the mean and scaled results are equidistant from the ground truth (Figure 1C, right box). Therefore, PERCEPT is well-placed to provide a value that captures both the effect size and the confidence with which it differs from the ground truth.

**Figure 2:**
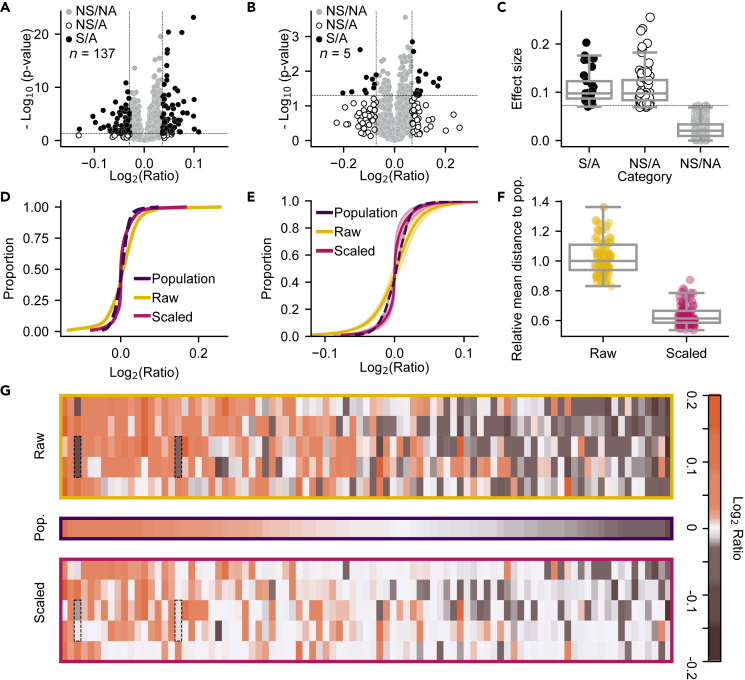
PERCEPT applied to small*-n* proteomics datasets results in better approximation of the ground truth (A and B) Volcano plots for (A) complete large*-n* cohort (n = 137) and (B) exemplar small*-n* subsampled dataset (n = 5). The threshold for significance was assigned as *p* < 0.05 (-log_10_(*p-*value) > 1.3; horizontal dotted lines). Thresholds for effect size were calculated as the 5th and 95th percentiles (vertical dotted lines) of the dataset. The mean for each quantified protein is shown (dots) colored according to whether it is considered non-significant and non-affected (NS/NA; gray), non-significant and affected (NS/A; open black circles) or significant and affected (S/A; closed black circles) based on its position relative to these thresholds. (C) The mean absolute effect size for individual proteins in each category in B. The mean threshold is also shown (dotted line). (D) Cumulative distribution of exemplar dataset presented in B before (Sample; yellow) and after (Scaled; magenta) PERCEPT, compared to the ground truth (Population; purple). PERCEPT was applied to the mean log_2_ abundance ratio for each protein using the corresponding *p*-value derived from a one-sample t-test against the hypothetical mean of 0 and a penalty factor of 20. (E) Mean cumulative distribution for randomly sampled small*-n* datasets, presented as in D. Shown is mean ± S.D. for the fitted cumulative distribution across all proteins in 100 trials where the biological replicates have been randomly subsampled (n = 5), see STAR methods for detailed fitting and averaging techniques. (F) Average distance of each small*-n* dataset (dots) from the population was assessed before (Raw) and after (Scaled) scaling via the absolute difference (sample – population). (G) Heatmap featuring 100 randomly selected proteins from the nonsignificant-affected category. Each row contains the mean for individual proteins in one of five exemplar small*-n* datasets, either before (Raw) or after (Scaled) scaling, compared to the population value for these proteins (Pop.). Dotted boxes outline two examples of proteins which are drawn toward the population value after scaling. In C, F, boxplots are displayed as follows: center line corresponds to the median; box limits display upper and lower quartiles; and where shown whiskers extend to the last or first data point that is within 1.5× the interquartile range of the box limits in the upper and lower directions, respectively. See also Figures S2–S4.

Comparative omics studies generate datasets that can particularly suffer from binary *p*-value thresholding. The datasets are usually large, with low replicate number and hence have high variability. To illustrate this, we analyzed a large multi-center cohort study on Alzheimer’s disease (AD), focusing on the analysis of cerebrospinal fluid (CSF) through proteomics. The study encompassed a total of 137 donors.^10^ Volcano plots, which are scatterplots of effect size (e.g., fold-change in protein abundance) plotted against significance (e.g., inverse log-transformed *p-*value), are commonly used to visualize and enable selection of the data points of significance^11^ (Figure 2A). In traditional analyses, data points need to meet two criteria for significance, a user-defined threshold for effect size (often >1.5-fold change), and a *p*-value. We will call datapoints that meet these criteria the significant, affected (SA) group – these are the proteins normally taken forward for analyses to determine their relevance to the scientific question at hand. In contrast, we refer to the proteins that meet only the effect size threshold as the nonsignificant, affected group – represented by the lower left and right quadrants of a volcano plot (Figure 2A; nonsignificant, affected (NSA)). In cohorts with many participants such as this, only a small fraction of proteins is categorized as nonsignificant but affected (Figure 2A; 1.3%). Nonetheless, the result of traditional analyses is these potentially interesting datapoints being excluded.

The challenge of excluding potentially interesting datapoints grows as sample size decreases. We simulated a smaller*-n* dataset by randomly selecting only 5 donors from the original study. In this reduced dataset, the percentage of proteins falling into the nonsignificant, affected category increased more than 4-fold, reaching 5.6% (Figure 2B). These datapoints are lost to further analyses despite often exhibiting effect sizes as large, or larger than, those deemed of interest by binary significance thresholding (Figure 2C).

PERCEPT scaling of this smaller-*n* dataset was able to align the distribution of protein abundances more closely with the overall population than the raw abundance distribution (Figure 2D). In this instance, PERCEPT was applied to the mean log_2_ abundance ratio for each protein using *p*-values derived from a one-sample t-test of log_2_ abundance ratios for that protein against the hypothetical mean of 0. Consistent with our initial application of this method to ‘omics data,^12^ scaling was performed using a penalty factor of 20. The enhanced alignment of scaled data to the ground truth was consistently observed across 100 repeated variations of the small*-n* subsampling (Figures 2E and 2F). This was quantitatively evaluated in two ways. First, by computing the mean difference between the population value and the raw or scaled value for individual proteins in each of the 100 small*-n* datasets. The scaled dataset was, on average, 39% closer to the population as compared to the raw dataset (Figure 2F). Second, a Bland-Altman plot^13^ was prepared, which visualizes the mean difference between two measures compared to the mean of those measures (Figure S2). This visualization revealed the scaled values are on average closer to the population (Figure S2B; mean difference 0.0003 with 95% C.I. at 0.026, −0.025) when compared to the corresponding raw values (Figure S2A; mean difference 0.0008 with 95% C.I. at 0.047, −0.045). This reduction in distance from the population is also visually apparent when examining the sub-sampled datasets before and after scaling using a heatmap (Figure 2G).

These findings demonstrate that PERCEPT can circumvent the need for discarding individual proteins based on weak statistical measures caused by variability while enhancing the alignment of the dataset as a whole to the ground truth. Similar observations were made when comparing pairs of small*-n* datasets, where the scaled values exhibit a stronger correlation across studies investigating the same disease state after scaling (Figure S3). Further analyses on different biomarker data also revealed similar findings (Figure S4). Together, these applications demonstrate the applicability and exquisite tunability of PERCEPT for datasets from diverse methodologies.

## Discussion

PERCEPT provides an alternative to binary statistical thresholding. At its core, PERCEPT is a simple to use addition to the standard statistical testing workflow which avoids the pitfalls of binary thresholding of *p*-values. Doing so enables life scientists to capture a holistic view of their data, creating space for more nuanced patterns to emerge without the substitution of standard statistical approaches for complex alternative tools. Omics data are particularly ripe for PERCEPT because replicate numbers are usually low, and data is often highly variable. This means a higher number of replicates than what is usually practicable to collect is needed to reach the significance threshold for even moderately affected datapoints.^1^ In clinical omics studies, obtaining a sufficient number of replicates can be especially difficult due to the high heterogeneity among human cohorts.^14^ This limitation can result in a large portion of affected but nonsignificant candidates being overlooked.

The application of PERCEPT requires a known hypothetical value ($m_{0}$) from which a statistical test can measure the deviation of sample values and against which PERCEPT scaling is performed. For most proteomics and transcriptomics data, this is not a challenge because the data is usually reported in a normalized manner, where $m_{0}=1\;or\;0$. The other real datasets utilized in this manuscript came from a different technology; namely, ELISA-based biomarker discovery studies, in which ratios were created by normalizing the disease cohorts to their corresponding controls. $m_{0}=1$ was also the case for these data. For these applications, the statistical test of choice is a one-sample t-test. While far less common in real experimental settings, PERCEPT can be equally applied to non-ratiometric datasets provided a known hypothetical value is available (Figure 1B, Figure S1B). Similarly, *p*-values may be derived from other statistical tests.

One consequence of PERCEPT is a loss of “certainty” regarding which data points are considered meaningful solely based on statistical significance, shifting this responsibility heavily onto effect size. However, as indicated earlier in the manuscript, statistical inferences based solely on a binary threshold *p*-value should be further investigated anyway before making scientific conclusions. It remains crucial to differentiate meaningful data points, since focusing on a smaller set of variables facilitates linking cause and effect and increases the likelihood of identifying consequential aspects of the biological process under investigation.^15^ While using a standard effect size is an option, such as the 2-fold change commonly used in proteomics experiments, we advocate for data-driven thresholding of effect size. This may present challenges for researchers; however, we argue that it is ultimately beneficial as it requires researchers to establish effect size thresholds that are relevant to their specific experimental context. In this study, we employed a method to measure the pseudo-variation of control samples through randomization,^12^ although alternative approaches such as percentiles or z-scores are also available.^2^

Approaches also exist which effectively combine data scaling and selection, in both frequentist (least absolute shrinkage and selection operator (LASSO; L1), or ridge (L2) regularization) and Bayesian statistics. For example, LASSO determines a set of coefficients by adding a penalty term to the linear regression function where sparsity (i.e., and abundance of 0 coefficients that effectively remove the feature from consideration) is encouraged by setting large penalty terms. These techniques were recently compared using a proteomics dataset, alongside traditional binary statistical thresholding.^16^ Given the differing philosophies to data grouping underlying each of these methods, it is perhaps unsurprising that each approach yielded a different list of proteins of interest. Similarly, when applied to our small-*n* subsampled datasets, proteins identified by LASSO or ridge regularization as having non-zero coefficients were often those which would not meet the traditional statistical significance threshold (Figures S2C–S2F; small colored markers). This is despite often having a large effect size according to the ground truth. Proteins identified by these regularization approaches often retained effect sizes closely linked to their ground truth counterparts after PERCEPT scaling, found in the lower left and upper right quadrants (Figures S2D and S2F; colored markers). Importantly, PERCEPT also retains proteins with relevant (true) effect sizes that were not identified by regularization or kept via the traditional binary significance thresholding approach (Figures S2D and S2F; small gray markers).

In summary, PERCEPT provides researchers with a powerful, flexible addition to their analytical toolbox for data-driven and nuanced interpretation of their datasets. Such methods are becoming essential as various fields generate increasingly deep datasets that cannot be matched by the breadth of replicates needed to address the inherent variability in biological systems. Most compelling is the fact that these approaches are user-friendly and accessible, making them easily adaptable for non-experts. This means that researchers from diverse fields can quickly and effectively incorporate PERCEPT into their investigations. By embracing this methodology, researchers can unlock the full potential of their data, enabling discoveries across a wide range of disciplines.

### Limitations of the study

PERCEPT scaling is designed for studies in which sample sizes are limited. By design, ‘significant’ *p*-values produce a scaling factor that does not substantially alter the original value (Figure S1A), being at least ∼0.7 even when the penalty factor is very large (*F* = 500). Given the inherent link between large sample sizes and significant *p*-values, with a sufficiently large sample a statistical test will almost always demonstrate a significant difference.^17^ Thus, the application of PERCEPT to large sample sizes is unlikely to differ notably from mean smoothing.

Another important point to note is that PERCEPT scaling flattens the effect sizes (with the extent depending on the scaling factor *F*), so the magnitude of PERCEPT-scaled data may not always be directly equivalent to the true effect size.

## STAR★Methods

### Key resources table

**Table:** 

REAGENT or RESOURCE	SOURCE	IDENTIFIER
**Deposited data**		

**Omics** - Bader	Molecular Systems Biology	https://doi.org/10.15252/msb.20199356 Filenames: msb199356-sup-0004-DatasetEV2.xlsx; msb199356-sup-0005-DatasetEV3.xlsx
**Omics -** Bai	Neuron	https://doi.org/10.1016/j.neuron.2019.12.015 **Filenames:** NIHMS1569542-supplement-4.xlsx
**Omics -** Bereman	Scientific Reports	https://doi.org/10.1038/s41598-018-34642-x **Filenames:** 41598_2018_34642_MOESM1_ESM.xlsx
**Omics -** Collins	Journal of Proteome Research	https://doi.org/10.1021/acs.jproteome.5b00804 **Filenames:** NIHMS898604-supplement-Table_S-1.xlsx
**Omics -** DAlessandro	Journal of Proteome Research	https://doi.org/10.1021/acs.jproteome.0c00365 Filenames: pr0c00365_si_002.xlsx
**Omics -** Di	Signal Transduction and Targeted Therapy	https://doi.org/10.1038/s41392-020-00333-1 Filenames: 41392_2020_333_MOESM2_ESM.xls
**Biomarkers -** Bourbouli	Dementia and Geriatric Cognitive Disorders	https://doi.org/10.1159/000478979 Figure 1
**Biomarkers -** Hosokawa	International Journal of Neuroscience	https://doi.org/10.3109/00207454.2013.848440 Figure 2
**Biomarkers -** Kasai	Acta Neuropathologica	https://doi.org/10.1007/s00401-008-0456-1 Figure 2
**Biomarkers -** Kuiperij	Journal of Alzheimer’s Disease	https://doi.org/10.3233/JAD-160386 Figure 2
**Biomarkers -** Noto	Amyotrophic Lateral Sclerosis	https://doi.org/10.3109/17482968.2010.541263 Figure 1
**Biomarkers -** Suarez-Calvet	Journal of Neurology, Neurosurgery & Psychiatry	https://doi.org/10.1136/jnnp-2013-305972 Figure 2
Simulated & pre-processed datasets	This manuscript	Zenodo: https://doi.org/10.5281/zenodo.8127985

**Software and algorithms**		

Custom analysis scripts	This manuscript	Zenodo: https://doi.org/10.5281/zenodo.8128038
*WebPlotDigitizer* v4.7	Automeris	https://apps.automeris.io/wpd/

### Resource availability

#### Lead contact

Further information and requests for resources should be directed to and will be fulfilled by the lead contact, Dezerae Cox (dezerae@uow.edu.au).

#### Materials availability

This study did not generate new unique reagents.

#### Data and code availability

• This paper analyzes existing, publicly available data. These accession numbers for the datasets are listed in the key resources table. Simulated datasets and intermediate, preprocessed datasets have been deposited at Zenodo and are publicly available as of the date of publication. DOIs are listed in the key resources table.
• All original code has been deposited at Zenodo and is publicly available as of the date of publication. DOIs are listed in the key resources table.
• Any additional information required to reanalyze the data reported in this paper is available from the lead contact upon request.

### Method details

#### PERCEPT scaling

The scaled value is calculated according to Equation 1:

(Equation 1) $$V=m_{0}+\frac{m_{0}-m_{1}}{-F^{p}}$$

where V corresponds to the scaled value, $m_{0}$ corresponds to the hypothetical mean, $m_{1}$ corresponds to the mean of the replicate measurements, F corresponds to the penalty factor and p corresponds to the -value. The *p-*value may be derived from a statistical test appropriate for an individual experimental circumstance, but in our hands is usually derived from a one-sample t-test of the replicate measurements against the hypothetical mean value ($m_{0}$). The penalty factor F allows the weight of the scaling factor ($-\frac{1}{F^{p\;}}$) to be fine-tuned, such that larger F values more strongly scale the replicate mean toward the hypothetical (Figure S1). We recommend F=10×n as a reasonable starting point for tuning.

#### Simulated image dataset

Simulated image data were generated using the python package NumPy.^18^ The binary ground truth image data was created from an array consisting of 0’s and 1’s. To each x, y coordinate a random noise value drawn from the normal distribution was added. This was repeated 100 times to generate the large*-n* sample set. From this population a random selection of 5 images was collected as the small*-n* dataset. Finally, PERCEPT was applied to each set of pixel values in this small*-n* dataset against the ground-truth value with a penalty factor of 20 to produce the scaled value.

#### Large*-n* proteomics dataset

While it remains unfeasible to sample the entire human population, and thus to derive an actual ground truth value for human characteristics, we reasoned ‘pseudo’ ground truth values could be compiled from studies containing in excess of 100 participants. We identified a recent large multi-center Alzheimer’s disease study^10^ which satisfied this criteria (n = 137) and reported raw values for each individual donor. While this is not strictly required for the application of PERCEPT (for which only the *p*-value, sample mean and *n* are needed), the availability of raw data in this instance allowed us to compare large-*n* and small-*n* datasets drawn from the same study (see subsampling small-n proteomic datasets below).

Raw per-protein abundance values were extracted for each donor from the published supplementary material “MSB-19-9356_Dataset EV3”.^10^ The provided protein identifiers were first mapped to standard UniProtKB accessions via the UniProt API, which also removed unreviewed or ambiguous entries. For data derived from each center, the change in abundance for individual proteins for each donor was calculated as the reported intensity value divided by the mean intensity of that protein in the control cohort. In addition, the control cohort was collected and compared directly against a randomized version of itself to define the pseudo-control ratios. These ratios capture some of the inherent experimental variability, such that data-driven thresholds can be established which encompass 90% of this pseudo-control distribution. The resulting thresholds delineate the absolute minimum abundance ratio considered to be biologically of interest. This approach experimentally determines the threshold values in contrast to the standard threshold of |log_2_ Fold Change| > 1.

Finally, the calculated abundance ratios for the AD cohort across all centers were subjected to a one-sample t-test against the hypothetical value of 1, and the resulting *p-*values used for thresholding of the traditional volcano plot (−log_10_(*p-*value) > 1.3). Based on the location of individual proteins relative to the abundance and *p-*value thresholds, three categories were defined; significant and affected (S/A; −log_10_(*p-*value) > 1.3, log_2_(abundance) > 0.077 or log_2_(abundance) < −0.058), non-significant and non-affected (NS/NA; −log_10_(*p-*value) < 1.3, log_2_(abundance) < 0.077 and log_2_(abundance) > −0.058), and non-significant affected (NS/A; -log_10_(*p-*value) < 1.3, log_2_(abundance) > 0.077 or log_2_(abundance) < −0.058). This dataset was then treated as the ground truth (Population) dataset.

#### Subsampling small*-n* proteomic datasets

Smaller*-n* datasets were simulated by randomly selecting 5 individuals from the AD cohort. The mean log_2_ abundance ratio per protein (Raw) was then optionally subjected to scaling (Scaled) with PERCEPT using a one-sample t-test against the hypothetical mean of 0 and a penalty factor F of 20. To compare the relative distance of the raw and scaled values from the population the mean of the absolute difference per protein was calculated, yielding a single distance value.

This subsampling process was then repeated 100 times, and the distance measures for both the raw and scaled datasets normalized to the mean distance of the subsampled raw datasets over the 100 trials. To enable comparison of the cumulative distributions between raw, scaled and population measurements across the 100 subsampling trials, it was necessary to first interpolate standard increments in the cumulative distribution. For each subsampled distribution, the cumulative distribution was calculated and the corresponding Log_2_(abundance) at a set of 100 equidistant increments from 0 to 1 (0, 0.01, 0.02, 0.03 … 0.99, 1) was interpolated using the ‘nearest’ method provided by pandas.^19^

Frequentist regularization methods were applied to the subsampled data using the python implementation of LASSO or ridge functionality provided by the scikit-learn package.^20^ Raw or PERCEPT scaled abundance ratios were used for each protein across the 5 subsampled individuals. The 50 most extreme non-zero coefficients were extracted from each method for comparison.

#### Comparative small*-n* proteomic datasets

We reasoned that, given the ability of the PERCEPT method to closer approximate the ground truth from small*-n* datasets, the agreement between small*-n* datasets sharing a ground truth should be improved following scaling. To examine this, we collected comparable small*-n* proteomic datasets from two additional disease contexts: amyotrophic lateral sclerosis (ALS)^21^^,^^22^ containing 32 and 9 samples respectively, and COVID-19^23^^,^^24^ containing 33 and 23 samples respectively. We also identified a small*-n* AD study^25^ containing 8 samples which was compared to the dataset from Bader and colleagues.^10^ These studies were selected on the basis that they reported raw protein abundance values for individual participants in both the disease and control cohorts measured via proteomics of matched biofluids (i.e., serum or CSF).

Raw per-protein abundance values were extracted from the published supplementary material for each article, then processed as described above for the *Large-n proteomics dataset*. To enable compilation of these studies, individual distributions were normalized to the maximum absolute value, resulting in each dataset being symmetrically scaled to a maximum range of −1 to 1 without disrupting the center of 0. PERCEPT was applied to the mean per-protein log_2_ abundance ratio using *p-*values derived from a one-sample t-test against the hypothetical value of 0 and a variable penalty factor F equivalent to 10×n where n is the number of individual participants in the disease cohort. Tuning the penalty factor in this way effectively offsets the natural decrease in *p*-value associated with increasing sample size alone,^4^ facilitating a comparison of the effect of PERCEPT across datasets with different sample sizes.

The correlation was assessed using the Pearson’s correlation coefficient (*R*), which was calculated between each pair of datasets using the raw or scaled log_2_(Ratio) values for proteins quantified in both datasets from the pair. This was then repeated for each dataset pair to yield five *R* values per dataset before and after scaling. The resultant *R* values were then binned according to whether the paired datasets were from the same disease (inside), or different diseases (outside). Datasets from different diseases (‘outside’) are not expected to share this common ground truth and therefore the scaling process is not expected to improve their correlation. In this way, ‘outside’ dataset pairs serve as a negative control. Finally, the average *R* value for each dataset inside and outside was compared before and after scaling for each disease category.

#### Biomarker datasets

We also sought a single-parameter dataset for which we could readily derive a pseudo-ground truth value from large*-n* replicates. Studies reporting enzyme-linked immunosorbent assay (ELISA) quantitation of fluid biomarkers in human disease are an excellent candidate, owing to three characteristics: (1) the relative availability and simplicity of commercially available ELISA kits for popular target proteins has resulted in multiple studies reporting a complementary measure for a single protein across many control versus disease cohorts, (2) data are often reported for each donor such that individual values can be compiled across multiple studies; and (3) they are usually large*-n*.

We selected studies which quantified the concentration of TAR DNA-binding protein 43 (TDP-43) in CSF among patients with two closely related disorders, ALS and Frontotemporal Lobar Dementia (FTLD). A meta-analysis of these efforts is beyond the scope of this study, however more comprehensive reviews are available.^26^ We compiled data from six studies for which a quantitative measure of total TDP-43 concentration in CSF was reported for either ALS- or FTLD-derived samples alongside healthy control donors.^27^^,^^28^^,^^29^^,^^30^^,^^31^^,^^32^ Any individual datapoints not available via the Supplementary Information were extracted from each study using the WebPlotDigitizer tool (https://automeris.io/WebPlotDigitizer).^33^ To enable cross-study comparison, measurements for individual ALS and FTLD donors were first normalized according to the mean TDP-43 concentration in the control cohort for each study (ΔTDP-43). The resultant ΔTDP-43 was compiled for all donors from each of the control, ALS, and FTLD cohorts, representing the large*-n* population for which the mean ΔTDP-43 was then considered the ground truth. The per-study means for each cohort (the small*-n* sample values) were then scaled against the hypothetical value of 1 (i.e., no difference due to disease) using a one-sample t-test and a variable penalty factor F equivalent to 10×n where n is the number of donors in each cohort. Tuning the penalty factor in this way effectively offsets the natural decrease in *p*-value associated with increasing sample size alone, facilitating a comparison of the effect of PERCEPT across datasets with different sample sizes.

### Quantification and statistical analysis

Statistical analyses were performed using the scipy module in python.^34^ All statistical details can be found in the corresponding figure legend or methods. For comparison to traditional *p*-value thresholding, the threshold of *p* = 0.05 was considered significant.

## Supplemental information

Document S1. Figures S1‒S4
