# Supplementary material for: PERCEPT: Replacing binary p-value thresholding with scaling for more nuanced identification of sample differences
Source: iScience. 2024 May 3;27(6):109891. doi: 10.1016/j.isci.2024.109891 (PMC11145341; doi:10.1016/j.isci.2024.109891)
Supplement: Document S1. Figures S1‒S4 [file mmc1.pdf]

iScience, Volume *27*

Supplemental information

PERCEPT: Replacing binary *p*-value thresholding with scaling for more nuanced identification of sample differences

Dezerae Cox and Danny M. Hatters

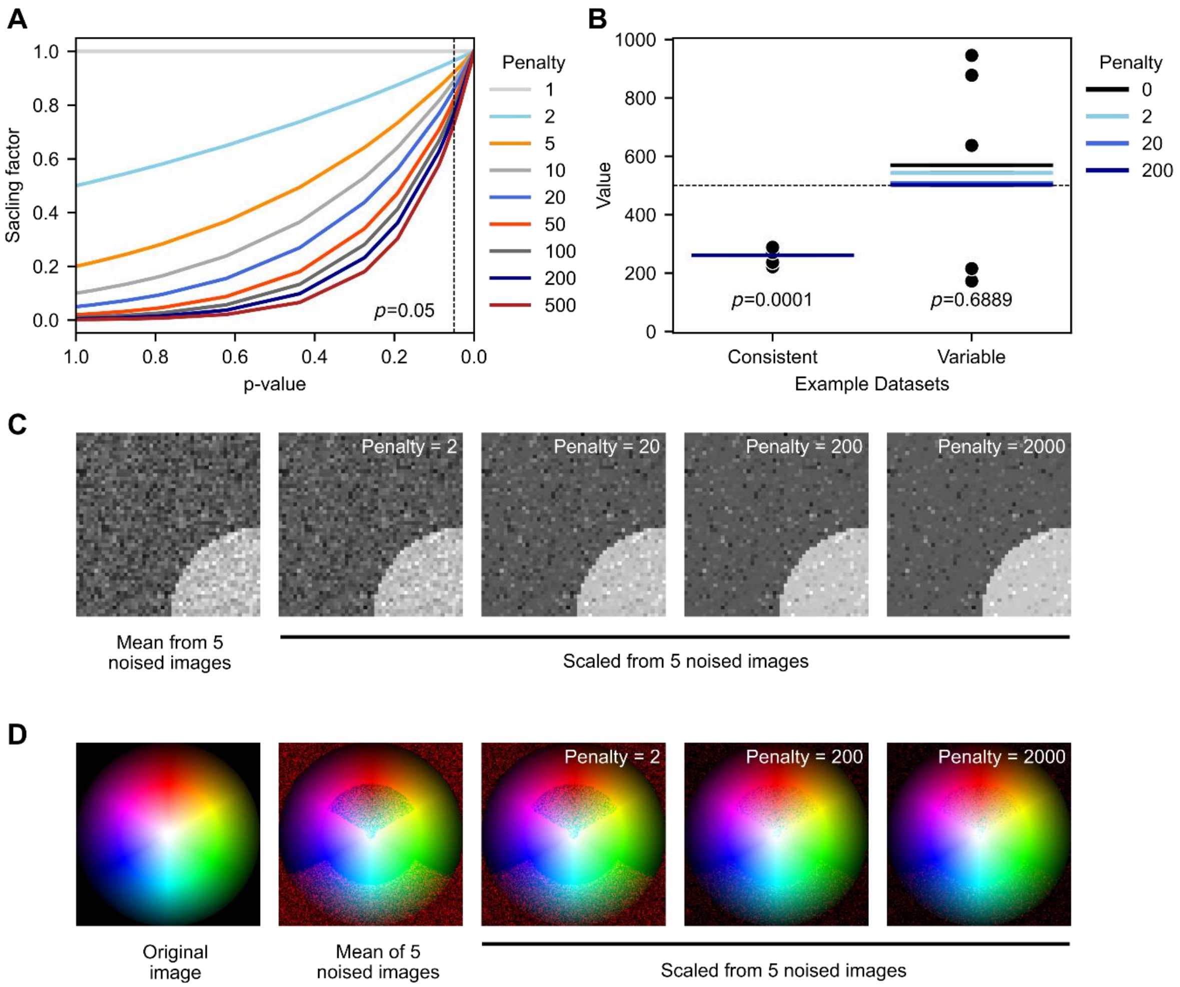

<u>Figure S1:</u> Incorporation of a penalty value enables fine-tuning of scaling strength, related to Figure 1. (A) Simulated *p*-values were converted to scaling factors using penalty values (F) ranging from 1 to 500. Increasing penalty weight decreases the scaling factor for a given *p*-value in a manner that is biased for larger *p*-values. **(B)** Scaling applied to simulated consistent (coefficient of variation < 0.3) and variable (coefficient of variation > 0.3) datasets (circles) using penalty values ranging from 0 to 200. PERCEPT was applied to the mean ratio for each dataset using the corresponding *p*-value derived from a one-sample t-test against the hypothetical mean of 500. The penalty value has negligible impact on the scaling factor for consistent datasets, whereas for variable datasets increasing the penalty value enhances scaling toward the hypothetical value (dotted line). **(C)** Visualisation of different penalty values when applied to simulated noisy image data. **(D)** Application of PERCEPT to simulated positive data in the form of the colorwheel dataset provided by skimage. Random noise was added to the red channel, then the mean of 5 such noised images was compared to the PERCEPT scaled version with a range of penalty factors.

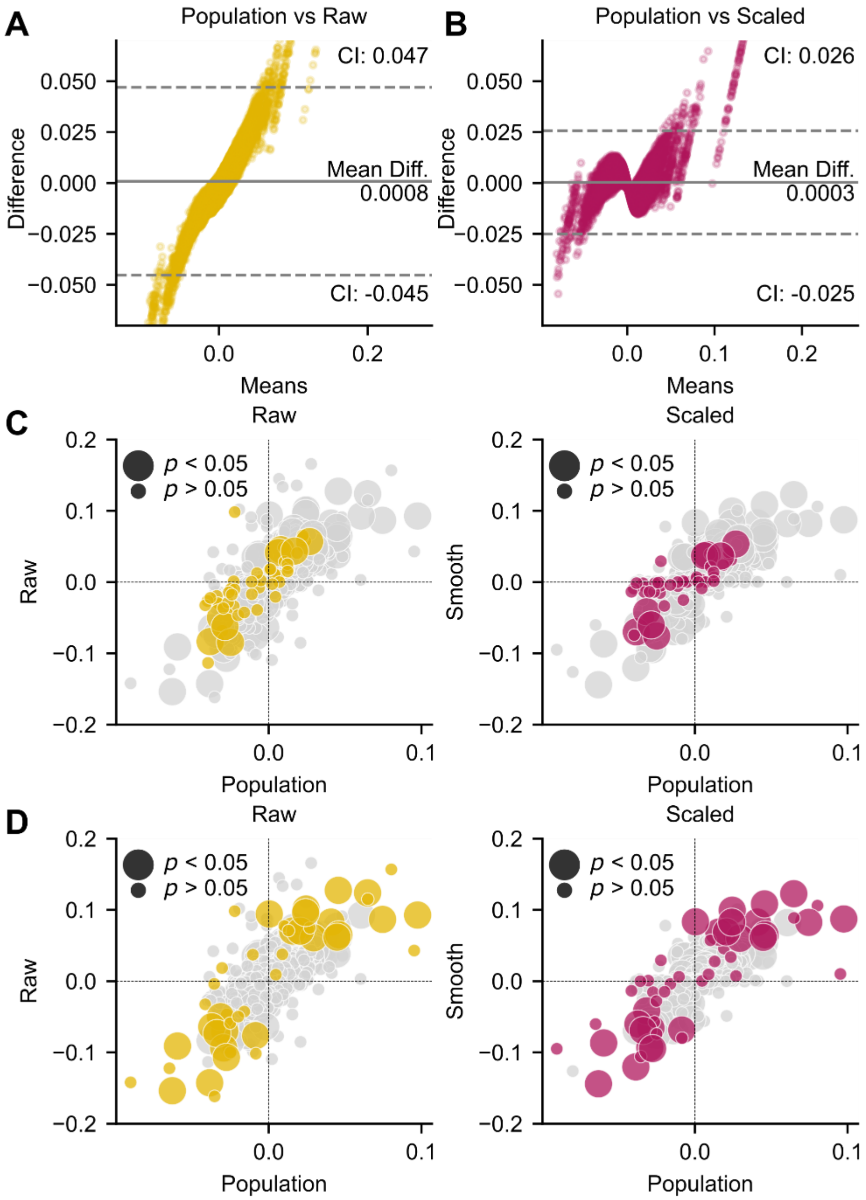

<u>Figure S2:</u> Benchmarking PERCEPT against the ground truth, and in contrast to other regularization methods, related to Figure 2. (A-B) Bland-Altman visualization of the subsampled small-*n* proteomics datasets. The mean of and difference between per-protein mean values is shown for each of the 100 simulated small-*n* trials. Shown is a scatterplot containing these 100 points for each individual protein for the **(A)** population vs raw, and **(B)** population vs scaled. In addition, for each comparison the mean difference (Mean Diff.; solid line) and 95% distribution intervals (CI; dotted lines) are annotated. **(C-D)** Comparison of **(C)** LASSO and **(D)** Ridge regularization with raw (left panels) or PERCEPT scaled (right panels) mean $\log_2$ protein abundances derived from one subsampled small-*n* dataset (*n*=5). Each protein is represented by a single marker, sized according to the p-value derived from traditional binary thresholding. Colored markers (yellow: raw; magenta; scaled) are those assigned the top 50 non-zero coefficients by regularization, and all other proteins are visualized in grey. Proteins in the upper right and lower left quadrants demonstrate agreement between PERCEPT and regularization methods aligned with the ground truth (colored), and proteins whose effect size is consistent with the ground truth and maintained by PERCEPT but not regularization (grey). Many of the latter would also be ignored following binary thresholding (small markers).

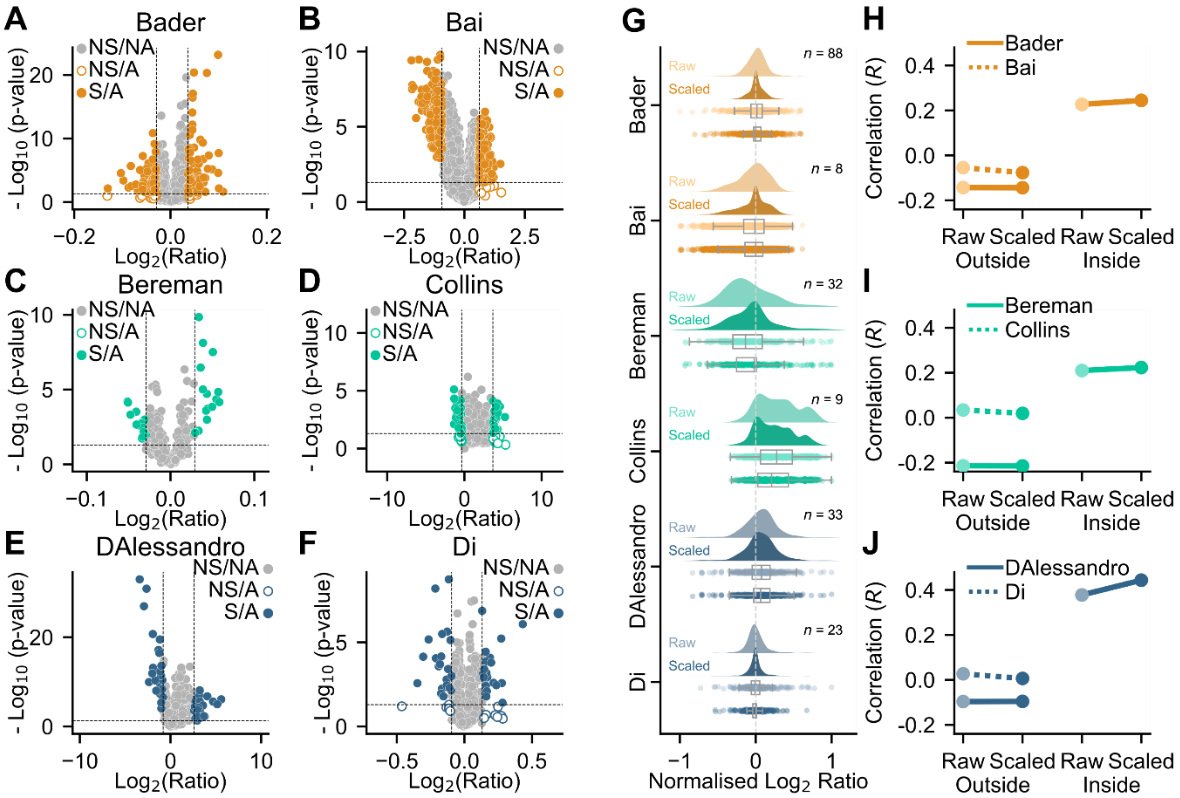

<u>Figure S3:</u> PERCEPT scaling of large-*n* proteomics datasets improves correlation across studies, related to Figure 2. (A-F) Quantitative proteomics datasets were collected from six studies reporting on Alzheimer's disease (AD; gold), amyotrophic lateral sclerosis (ALS; green), or COVID (blue) cohorts. Volcano plots are shown for each raw dataset, including consistent thresholds calculated for effect size as the 5 and 95 percentiles (vertical dotted lines) and significance as $p < 0.05$ ($-\log_{10}(p\text{-value}) > 1.3$; horizontal dotted lines). The mean for each quantified protein is shown (dots) colored according to whether it is considered non-significant and non-affected (NS/NA); non-significant and affected (NS/A) or significant and effected (S/A) based on its position relative to these thresholds. **(G)** To enable compilation of these studies, individual distributions were normalised to the maximum absolute value, resulting in each dataset being symmetrically scaled to a maximum range of -1 to 1 without disrupting the center of 0. The resultant distribution of protein values are shown before (Raw) and after (Scaled) scaling. PERCEPT was applied to the mean ratio for each protein using the corresponding *p*-value derived from a one-sample t-test against the hypothetical mean of 0 and a penalty factor equivalent to $n \times 10$. The number of biological replicates over which the mean was calculated for individual proteins is annotated. **(H-J)** The Pearson's correlation between each pair of datasets (A – F) was assessed before and after scaling. Correlation coefficients (*R*) were calculated using the $\log_2$(Ratio) values for proteins quantified in both datasets of the pair, a process repeated for each dataset pair to yield five *R* values per dataset before and after scaling. The resultant *R* values were then binned according to whether the paired datasets were from the same disease (inside), or different diseases (outside). While scaling is anticipated to improve the correlation between datasets from the same disease (inside) by bringing both datasets closer to the shared ground truth, 'outside' comparisons should not share the same ground truth and therefore represent a negative control. The average *R* value for each dataset inside and outside was compared before and after scaling for the (H) AD , (I) ALS and (J) COVID cohorts. In G, boxplots are displayed as follows: center line corresponds to the median; box limits display upper and lower quartiles; and where shown whiskers extend to the last or first data point that is within 1.5× the interquartile range of the box limits in the upper and lower directions, respectively.

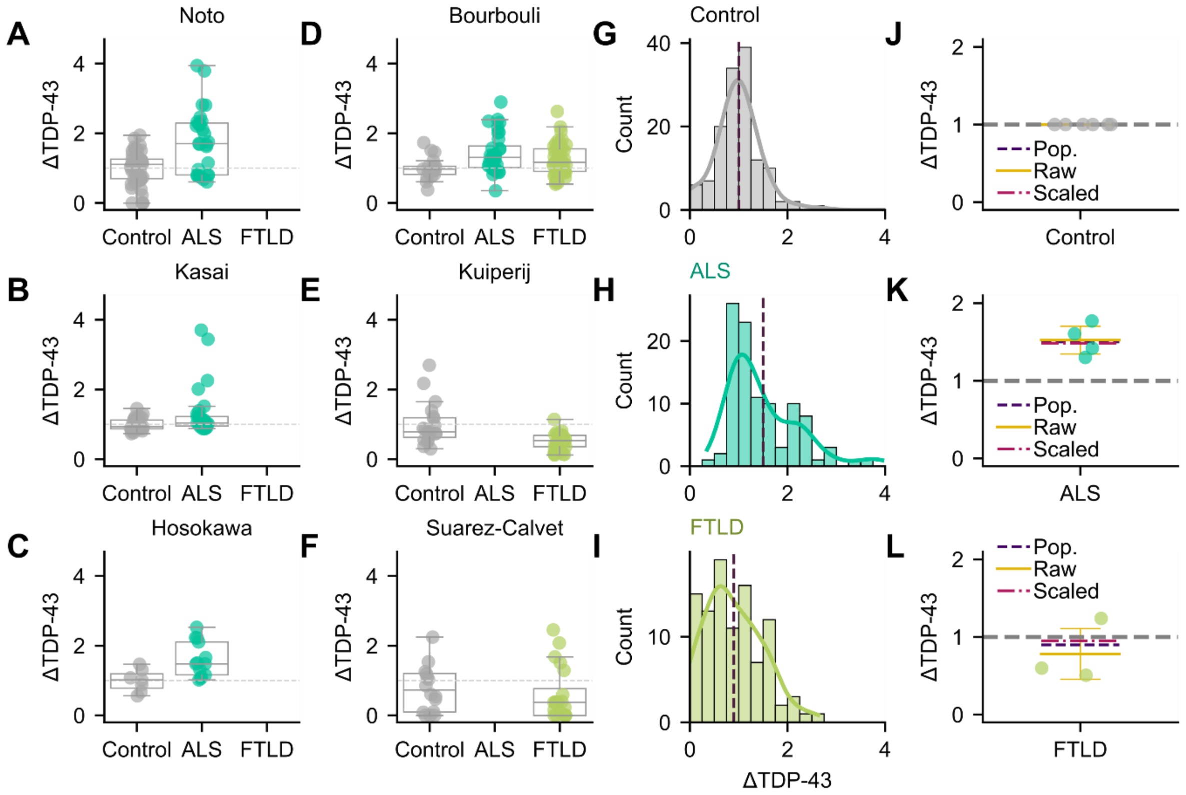

Figure S4: Application of PERCEPT scaling to single-parameter biomarker datasets, related to Figure 2. (A-F) Quantitative ELISA measurements of TDP-43 concentration in cerebrospinal fluid were collected from six studies reporting on ALS or FTLD cohorts. To enable compilation of these studies, individual data points were normalised to the mean control value for each study such that values for each control cohort are centred on 1 (dotted line). **(G-I)** Compiled normalised dataset distributions for combined (G) control, (H) ALS and (I) FTLD cohorts. Histograms are overlayed with gaussian kernel density estimates (solid lines) and the pseudo-population mean (dotted lines) for each cohort. **(J-L)** PERCEPT scaling of per-study sample measures. The mean ΔTDP-43 concentration for each study cohort in A-F was collected as a small-*n* dataset. The mean value for the individual (J) control, (K) ALS and (L) FTLD study cohorts was compared with the compiled population means shown in G-I (dashed line) before and after scaling. PERCEPT was applied using a one-sample t-test against the hypothetical mean of 1 (no difference to control) and a variable penalty factor equivalent to $n \times 10$. In A - D, data points for individual donors are overlayed on boxplots displayed as follows: center line corresponds to the mean; box limits display upper and lower quartiles; and where shown whiskers extend to the last or first data point that is within 1.5× the interquartile range of the box limits in the upper and lower directions, respectively. In J-L, individual sample datapoints (mean of each study cohort) are overlayed with mean ± S.D.
